# Na Era da Reabilitação Cardíaca, Ainda Existem Pacientes Portadores de Angina Refratária “Sem Opção”?: Um Relato de Caso

**DOI:** 10.36660/abc.20230007

**Published:** 2023-10-06

**Authors:** Luciana Oliveira Cascaes Dourado, Camila Paixão Jordão, Camila Regina Alves Assumpção, Luciana Diniz Nagem Janot de Matos

**Affiliations:** 1 Hospital das Clínicas Faculdade de Medicina Universidade de São Paulo São Paulo SP Brasil Instituto do Coração do Hospital das Clínicas da Faculdade de Medicina da Universidade de São Paulo , São Paulo , SP – Brasil; 2 Hospital Israelita Albert Einstein São Paulo SP Brasil Hospital Israelita Albert Einstein , São Paulo , SP – Brasil

**Keywords:** Angina Pectoris, Exercício, Reabilitação Cardíaca

## Abstract

A reabilitação cardíaca baseada em exercícios, um tratamento adjuvante eficaz e seguro recomendado para pacientes com doença arterial coronariana, é pouco aplicada em pacientes com angina refratária (AR) devido a dificuldades relacionadas à segurança, prescrição do treinamento e o seu manejo clínico. Este relato de caso apresenta um paciente “sem opção” com AR, incluído em um programa de exercícios de 12 semanas de duração, composto de 40 minutos de exercício aeróbico em esteira por sessão, três vezes por semana, e intensidade prescrita entre limiares isquêmicos/anginosos e limiar ventilatório 1, conforme obtidos no teste de exercício cardiopulmonar; angina leve a moderada foi permitida durante o treinamento. Além disso, foram realizados 15 minutos de treinamento de resistência de intensidade moderada (exercícios de grandes grupos musculares, duas séries de 8 a 12 repetições). Ao final do protocolo, o paciente apresentou melhora importante no desempenho funcional (VO _2_ máximo de 17,0 ml/kg/min para 27,3 ml/kg/min), limiar anginoso (FC de 68 bpm para 95 bpm) e na intensidade da dor torácica (nível 7 para 5) sem eventos clínicos adversos durante o período. A reabilitação cardíaca baseada em exercícios se mostrou segura, mesmo na ocorrência de angina/isquemia durante o treinamento, de acordo com a tolerabilidade aos sintomas e outros sinais clínicos de alerta.

## Introdução

A angina refratária (AR) é uma condição crônica caracterizada por angina debilitante não controlada pela combinação de tratamentos médicos em pacientes não elegíveis à revascularização coronariana, seja por doença coronariana difusa, ou por alto risco de complicações, ou mesmo ausência de doença coronariana epicárdica. ^1^

Esses pacientes, referidos como “sem opção”, frequentemente apresentam má qualidade de vida e depressão, ^2^ e seu tratamento passa a ser um desafio. Muitos tratamentos alternativos, alguns experimentais, foram propostos e os resultados são variáveis na melhora dos sintomas e da qualidade de vida. ^3 , 4^

A reabilitação cardíaca baseada em exercícios (RCE), um tratamento adjuvante eficaz e seguro recomendado para pacientes com doença arterial coronariana (DAC), ^4 , 5^ tem sido pouco aplicada em pacientes com AR ^6 , 7^ devido ao receio da ocorrência de eventos adversos durante o treinamento físico, uma vez que esses pacientes apresentam baixo limiar isquêmico, tornando a prescrição de exercícios particularmente desafiadora. ^8^

Aqui, apresentamos o caso de um paciente com AR considerado “sem opção”, incluído em um protocolo clínico de RCE para pacientes com AR. O estudo foi aprovado pelo comitê de ética e pesquisa (CAAE: 24308213.7.0000.0068), enviado e aprovado em Ensaios Clínicos (NCT03218891). As investigações seguiram a Declaração de Helsinque. O paciente deu seu consentimento informado por escrito.

### Apresentação do caso

**Figura 1:**
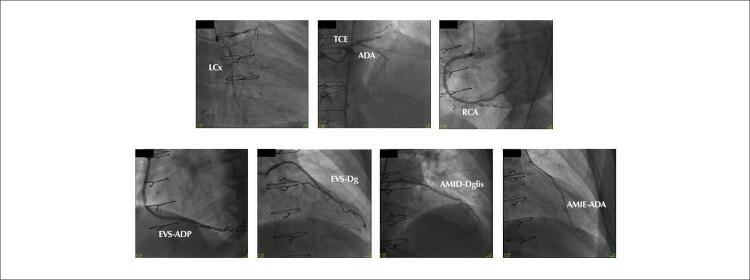
– Angiografia coronária demonstrando doença arterial coronariana difusa em paciente com angina refratária. LCx: ramo circunflexo esquerdo; TCE: tronco da coronária esquerda; ADA: artéria descendente anterior esquerda; EVS: enxerto de veia safena; ADP: artéria descendente posterior; Dglis: ramus diagonalis; Dg: ramo diagonal; AMID: artéria mamária interna direita; AMIE: artéria mamária interna esquerda.

Paciente do sexo masculino, 52 anos, apresentava DAC estável com angina progressiva debilitante de limiar variável, ocorrendo em repouso uma vez por semana, com necessidade de uso de nitrato sublingual, e em atividades cotidianas, apesar de terapia medicamentosa otimizada incluindo aspirina, 80 mg de atorvastatina, 10mg de Ezetimibe, 25 mg de Atenolol duas vezes ao dia, 5 mg de Amlodipina duas vezes ao dia, 20 mg de Mononitrato de isossorbida duas vezes ao dia e 35 mg de Trimetazidina duas vezes ao dia. O paciente apresentava o diagnóstico de hipercolesterolemia familiar e, há seis anos, foi submetido à cirurgia de revascularização miocárdica (CRM) associada à terapia celular ou placebo em um ensaio clínico projetado para pacientes com angina e DAC difusa, em que o território coronariano foi considerado desfavorável à CRM convencional. Persistiu assintomático por dois anos após a intervenção quando voltou a apresentar angina. Na ocasião, foi realizada cineangiocoronariografia ( Figura 1 ), evidenciando enxertos pérvios e DAC multiarterial difusa. O exame clínico revelou ausência de anormalidades ( Tabela 1 ) e índice de massa corporal (IMC) de 27,6 kg/m ^2^ . Os achados laboratoriais são mostrados na Tabela 1 . Foi realizado ecocardiograma de esforço, demonstrando fração de ejeção normal e isquemia dos segmentos ântero-apical e septal-apical.

**Tabela 1:** – Evolução do paciente entre a avaliação inicial e final do programa de reabilitação cardíaca baseado em exercícios de 12 semanas

Dados do paciente	Inicial	Final
**Achados clínicos**		
PAS (mmHg)	120	110
PAD (mmHg)	85	78
FC (bpm)	51	50
CA (cm)	95	96
Peso (kg)	81,6	81,7
**Achados laboratoriais**		
Hemoglobina (mg/dl)	15,7	15,8
Creatinina (mg/dl)	0,8	0,8
Glicose em jejum (mg/dl)	99	97
Hemoglobina glicosilada (%)	5,5	5,4
LDL-c (mg/dl)	101	93
HDL-c (mg/dl)	43	39
Triglicerídeos (mg/dl)	95	79
**Achados do TECP**		
Carga de trabalho máxima (%)	16	24
VO _2_ máximo (L/min)	1,3	2,2
VO _2_ máximo (mL/kg/min)	17,0	27,3
FC LT1 (bpm)	75	81
Pulso de O _2_ (mL/batimento)	17,1	18,9
Limiar de angina (bpm)	68	95
Classificação da angina (escala)	7	5

PAS: pressão arterial sistólica; PAD: pressão arterial diastólica; FC: frequência cardíaca; CA: circunferência abdominal; TECP: teste de exercício cardiopulmonar; LV1: limiar ventilatório 1.

O paciente foi encaminhado para RCE em um hospital terciário com experiência em AR. Foi realizado teste de exercício cardiopulmonar (TECP) inicial em esteira (2,5 mph + 2% a cada minuto) ( Tabela 1 ) e não foram encontradas anormalidades eletrocardiográficas durante o exercício.

O programa de RCE foi realizado em um centro de reabilitação cardiovascular de um hospital terciário, dentro de uma sala de treinamento com temperatura controlada, equipada com bicicletas, esteiras, aparelhos de musculação, pesos livres, colchonetes, bolas e faixas, contando também com o auxílio de profissionais de reabilitação qualificados (médico, profissional da educação física, fisioterapeuta).

A RCE incluiu 36 sessões de exercícios, ao longo de 12 semanas (três vezes por semana). As sessões consistiam em um total de 40 minutos de exercícios aeróbicos: 5 minutos de aquecimento, 30 minutos de exercício aeróbico contínuo em esteira à FC prescrita entre o limiar isquêmico/anginoso e o limiar ventilatório 1 (LV1) obtido no TECP, correspondendo a uma FC entre 68 a 75 bpm, e 5 minutos de desaquecimento. O exercício contínuo foi recomendado; no entanto, breves interrupções ou reduções na intensidade foram permitidas caso o paciente apresentasse angina moderada. O exercício era reiniciado ao desaparecerem os sintomas. O paciente foi continuamente monitorado por telemetria. Cinco miligramas de dinitrato de isossorbida sublingual eram administradas conforme necessário. Após o exercício aeróbico, foram realizados 15 minutos de exercícios de resistência de grandes grupos musculares (duas séries de 8 a 12 repetições) a 65% da carga máxima por repetição, medida pela escala de percepção de resistência OMINI (4 a 6 níveis). ^9^ O paciente foi instruído a expirar durante a fase concêntrica, inspirar durante a fase excêntrica e evitar a manobra de Valsalva.

**Figura 2:**
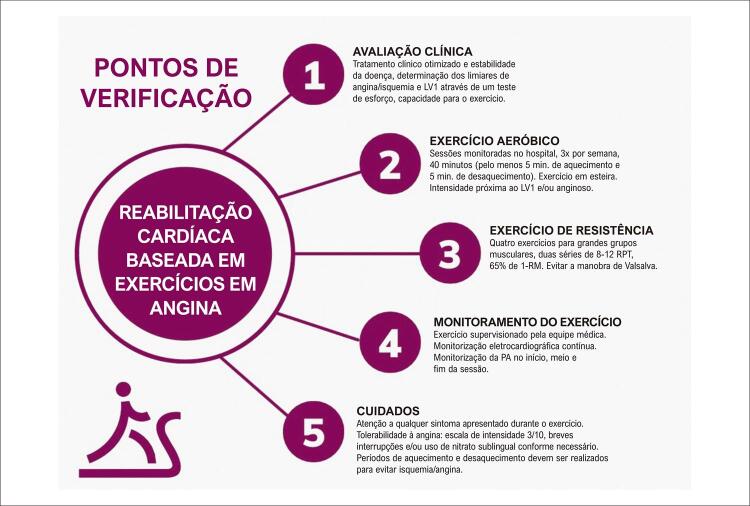
– Pontos de verificação para uma reabilitação cardíaca segura baseada em exercícios em pacientes com angina refratária. RPT: repetições; 1- RM: carga máxima para uma repetição; LV1: limiar ventilatório 1; PA: pressão arterial.

Uma escala visual numérica de dor ^6^ foi adotada e utilizada para determinar a interrupção do exercício ou reduzir sua intensidade. A escala foi graduada de 0 (sem dor) a 10 (dor intensa). A dor avaliada até 3 (leve a moderada) foi considerada o nível máximo de tolerabilidade permitido para o exercício aeróbico contínuo. Quando a dor atingia intensidade superior a 3 (moderada), o exercício era interrompido ou sua intensidade era diminuída. A pressão arterial (PA) foi medida antes, durante e após as sessões de exercício. A Figura 2 demonstra o protocolo de RCE, assim como o monitoramento e os cuidados com a angina.

Ao longo do programa de RCE de 12 semanas, o paciente completou 28 (78% de adesão) sessões de exercícios. Apesar da prescrição, a FC média do treinamento aeróbico foi de 81 bpm, já que o paciente tolerou uma maior intensidade de exercício aeróbico. A intensidade inicial da esteira foi fixada na velocidade de 3,0 km/h com 1% de inclinação, evoluindo para 4,5 km/h com 2% de inclinação ao final do programa. O paciente apresentou angina em 12 sessões de exercícios (sete episódios no primeiro mês, quatro no segundo e um no terceiro) graduada de 2 a 4, com FC média de 77 bpm, sem necessidade de uso de nitratos sublinguais nem interrupção de exercícios. A PA média foi de 105/70 mmHg no início, 120/70 mmHg durante e 125/70 mmHg ao final da sessão de exercício.

Após a conclusão do programa de exercícios, embora seu tratamento farmacológico permaneceu inalterado, seus sintomas foram significativamente reduzidos, apresentando apenas um episódio de angina em repouso nas últimas quatro semanas e sem angina durante a realização de suas atividades. Não houve alteração nos parâmetros do exame clínico.

O TECP final revelou aumento de 60% do VO _2_ máximo, aumento de 27bpm do limiar de angina e diminuição da intensidade da angina de 7 para 5. Não foram observadas anormalidades eletrocardiográficas durante o teste de esforço.

O paciente continuou praticando exercícios três vezes por semana durante os dois anos seguintes ao término do programa hospitalar, com melhora progressiva dos sintomas e da qualidade de vida.

## Discussão

Uma importante melhora clínica de um paciente com AR considerado “sem opção” foi relatada após um programa individualizado de RCE de 12 semanas, executado com segurança, apesar do treinamento de exercícios aeróbicos ter sido realizado acima do limiar de isquemia/angina obtido no TECP, o que reforça a importância dessa parte adjuvante, mas essencial, do manejo clínico desses pacientes.

A RCE, embora recomendada em algumas diretrizes, ^8^ raramente é aplicada em pacientes com AR devido às poucas evidências de segurança, eficácia e peculiaridades quanto à sua aplicação prática. Ela foi estudada nessa população pela primeira vez por Asbury et al., ^6^ demonstrando melhora na duração do *Progressive Shuttle Walking Test* , ansiedade e percepção de angina em um programa de reabilitação de oito semanas. Mais recentemente, Montenegro et al. ^7^ também demonstraram a segurança do exercício em pacientes com AR, já que uma sessão aeróbica de 40 minutos, mesmo na presença de angina, não aumentou a troponina-us e, portanto, não provocou lesão miocárdica.

Da mesma forma, Corre et al. ^10^ relataram o caso de um paciente com AR que treinou com base em um protocolo de 20 sessões com exercícios aeróbicos intervalados de alta intensidade, incluindo isquemia intermitente, e apresentou melhora importante dos sintomas anginosos e do limiar isquêmico. Tal estratégia demonstrou ser segura previamente ^11^ em 11 pacientes com cardiopatia isquêmica.

Vale ressaltar que os episódios de angina durante o treinamento físico, quando ocorridos, foram a uma FC superior ao limiar de angina do TECP. Essa diferença pode ser justificada, em parte, pelas diferentes condições impostas a um teste de exercício em laboratório e a um treinamento da vida real, como o incremento progressivo de carga que ocorre no TECP, somado à ausência de períodos de aquecimento e desaquecimento do teste. Assim, a FC do limiar de angina do TECP é uma variável importante para orientar a prescrição de exercícios aeróbicos, mas não uma limitação para o treinamento na vida real.

Portanto, a RCE se mostrou uma estratégia de tratamento segura e eficaz para este paciente com AR, apesar da ocorrência de angina/isquemia durante o treinamento, respeitando a tolerabilidade do paciente aos sintomas e outros sinais clínicos de alerta. É importante ressaltar que pacientes com AR apresentam características de alto risco para eventos induzidos pelo exercício. ^4 , 12^ Dessa forma, as condições apresentadas anteriormente, como estabilidade clínica, determinação de parâmetros clínicos individuais (limiar de angina e nível de tolerabilidade à angina) para a prescrição e orientação do treinamento, além da realização de RCE de forma supervisionada e em ambiente hospitalar, são necessários para a inclusão desses pacientes em programas de RCE.

Dadas as evidências atuais, é importante começarmos a considerar a RCE como parte integrante do tratamento desses pacientes. Além disso, estudos maiores e randomizados são necessários para reforçar essa indicação por meio de mais evidências de alta qualidade.
