# Supplementary material for: Na Era da Reabilitação Cardíaca, Ainda Existem Pacientes Portadores de Angina Refratária “Sem Opção”?: Um Relato de Caso
Source: Arq Bras Cardiol. 2023 Oct 6;120(9):e20230007. [Article in Portuguese] doi: 10.36660/abc.20230007 (PMC10567080; doi:10.36660/abc.20230007)
Supplement: Translation

# Translation: In the Cardiac Rehabilitation Era, is There a “No-Option” Refractory Angina Patient?: A Case Report

**Authors:** Luciana Oliveira Cascaes Dourado, Camila Paixão Jordão, Camila Regina Alves Assumpção, Luciana Diniz Nagem Janot de Matos

**Affiliations:** 1 Brazil Instituto do Coração do Hospital das Clínicas da Faculdade de Medicina da Universidade de São Paulo, São Paulo, SP – Brazil; 2 Brazil Hospital Israelita Albert Einstein, São Paulo, SP – Brazil

**Keywords:** Angina Pectoris, Exercise, Cardiac Rehabilitation

## Abstract

Exercise-based cardiac rehabilitation, an effective and safe adjuvant treatment recommended to patients with coronary artery disease, is scarcely applied to patients with refractory angina (RA) due to difficulties related to safety, trainning prescription and their clinical management. This case report presents an instance of a “no-option” patient with RA, who was included in a 12-week exercise program, in sessions consisted of 40 minutes of treadmill aerobic exercise, three times a week, and intensity prescribed between ischemic/angina threshold and ventilatory threshold 1, obtained in the cardiopulmonary exercise test; mild to moderate angina was allowed during training. Furthermore, 15 minutes of moderate-intensity resistance training (large group muscle exercises, two sets of 8 to 12 repetitions) was performed. At the end of the protocol, the patient presented an important improvement in functional performance (VO _2_ peak 17.0 ml/kg/min to 27.3 ml/kg/min), angina threshold (HR 68 bpm to 95 bpm), and intensity chest pain (levels 7 to 5) with no clinical adverse events during the period. Exercise-based cardiac rehabilitation was safe, even in the occurrence of angina/ischemia during training, according to tolerability to symptoms and other warning clinical signs.

## Introduction

Refractory angina (RA) is a chronic condition characterized by debilitating angina that is not controlled by the combination of medical treatment in patients not suitable to coronary revascularization either by a diffuse coronary disease, or high risk of complications, or even absence of epicardial coronary disease. ^1^

These patients, treated as “no-option,” frequently present a poor quality of life and depression, ^2^ and their treatment is a challenge. Many alternative treatments, some experimental, have been proposed, and the results are variable in improving symptoms and quality of life. ^3 , 4^

Exercise-based cardiac rehabilitation (ECR), an effective and safe adjuvant treatment recommended to patients with coronary artery disease (CAD), ^4 , 5^ has been scarcely applied to RA patients ^6 , 7^ by fear of adverse events during physical training since these patients present low ischemic threshold, making the prescription of exercises a challenge. ^8^

We present a case of a patient with RA considered as a “no-option,” included in a clinical protocol of ECR in patients with RA. The study was approved by the ethics and research committee (CAAE: 24308213.7.0000.0068), submitted to and approved in Clinical Trials (NCT03218891). Investigations followed the Declaration of Helsinki. The patient provided written informed consent.

### Case presentation

A 52-year-old male patient presented a stable CAD with debilitating progressive angina with variable threshold, occurring at rest once a week, with the need of sublingual nitrate use, and at ordinary activities, despite optimal medical therapy including Aspirin, Atorvastatin 80 mg, Ezetimibe 10mg, Atenolol 25 mg bid, Amlodipine 5 mg bid, Isosorbide mononitrate 20 mg bid, and Trimetazidine 35 mg bid. The patient had the diagnosis of familial hypercholesterolemia, and he was submitted six years ago to a coronary artery bypass graft (CABG) associated with cellular therapy or placebo in a clinical trial designed for patients with angina and diffuse CAD, in which a coronary territory was considered unfavorable to conventional CABG. He persisted asymptomatic for two years after the intervention when presented angina again. At that time, a coronary angiography ( Figure 1 ) was performed, showing previous grafts and diffuse multivessel CAD. Clinical examination revealed no abnormalities ( Table 1 ) and a body mass index (BMI) of 27.6 kg/m ^2^ . Laboratory findings are shown in Table 1 . An exercise echocardiogram was performed, demonstrating a normal ejection fraction and ischemia of anteroapical and septal apical segments.

**Figure 1:** – Coronary angiography demonstrating diffuse coronary artery disease in a patient with refractory angina. LCx: left circumflex branch; TLMCA: left main coronary artery; SVG: saphenous vein graft; PDA: posterior descending artery; Dglis: ramus diagonalis; Dg: diagonal branch; RIMA: right internal mammary artery.

**Table 1:** – Patient evolution from initial evaluation to the end of the 12-week exercise-based cardiac rehabilitation program

Patient data	Initial	Final
**Clinical findings**		
SBP (mmHg)	120	110
DBP (mmHg)	85	78
HR (bpm)	51	50
AC (cm)	95	96
Weight (kg)	81.6	81.7
**Laboratory findings**		
Hemoglobin (mg/dl)	15.7	15.8
Creatinine (mg/dl)	0.8	0.8
Fasting glucose (mg/dl)	99	97
Glycosylated hemoglobin (%)	5.5	5.4
LDL-c (mg/dl)	101	93
HDL-c (mg/dl)	43	39
Triglicerides (mg/dl)	95	79
**CPET findings**		
Maximal workload (%)	16	24
VO _2_ peak (L/min)	1.3	2,2
VO _2_ peak (mL/kg/min)	17.0	27.3
VT1 HR (bpm)	75	81
O _2_ pulse (mL/beat)	17.1	18.9
Angina threshold (bpm)	68	95
Angina grading (scale)	7	5

SBP: systolic blood pressure; DBP: diastolic blood pressure; HR: heart rate; AC: abdominal circumference; CPET: cardiopulmonary exercise test; VT1: ventilatory threshold 1.

The patient was referred to ECR at a tertiary hospital with experience in RA. An initial cardiopulmonary exercise test (CPET) on a treadmill (2.5 mph + 2% each minute) was performed ( Table 1 ), and no electrocardiographic abnormalities were found during the exercise.

The ECR program was performed in a tertiary hospital cardiovascular rehabilitation center, inside a temperature-controlled training room equipped with cycles, treadmills, strength exercise equipment, free weights, mats, balls, and bands, also assisted by qualified rehabilitation professionals (physician, physical education professionals, physiotherapist).

ECR included 36 exercise sessions, along 12 weeks (three times a week). Sessions consisted of a total of 40 minutes of aerobic exercises: 5 minutes of warm-up, 30 minutes of continuous aerobic exercise on a motorized treadmill at HR prescribed between ischemic/angina threshold and ventilatory threshold 1 (VT1) obtained in the CPET, corresponding to HR between 68 to 75 bpm, and 5 minutes of cool-down. Continuous exercise was recommended; however, brief interruptions or reductions in intensity were allowed if the patient experienced moderate angina. The exercise was restarted when the symptoms were no longer observed. The patients were continuously monitored by telemetry. A 5 mg of sublingual isosorbide dinitrate was administered as needed. After aerobic exercising, 15 minutes of large muscle groups resistance exercises were performed, on 65% 1-repetition maximum (two sets of 8 to 12 repetitions) measured from OMINI resistance perception scale (4 to 6 levels). ^9^ Subjects were instructed to exhale during the concentric phase, inhale during the eccentric phase, and not perform the Valsalva maneuver.

A visual numeric pain scale ^6^ was adopted and used either to determine exercise interruption or to reduce its intensity. The scale was graded from 0 (no pain) to 10 (severe pain). Pain rated up to 3 (mild to moderate) was considered the top tolerability level allowed for continuing aerobic exercise. When the pain reached intensity levels higher than 3 (moderate), exercise was interrupted, or its intensity was reduced. Blood pressure (BP) was measured before, during, and after exercise sessions. Figure 2 demonstrates the CR protocol, as well as angina monitoring and care.

**Figure 2:** – Checkpoints to a safe exercise-based cardiac rehabilitation in patients with refractory angina. RPT: repetitions; 1- RM: one-repetition maximum; VT1: ventilatory threshold 1; BP: blood pressure.

Along the 12-week ECR program, the patient completed 28 (78% adherence) exercise sessions. Despite the prescription, mean aerobic training HR was 81 bpm, since the patient tolerated higher aerobic exercise intensity. Initial treadmill intensity was set at a speed of 3.0 km/h with 1% incline, progressing to 4.5 km/h with 2% at the end of the program. He presented angina in 12 exercise sessions (seven episodes in the first month, four in the second, and one in the third month) graded 2 to 4, at 77 bpm mean HR, with no need for sublingual nitrates nor exercise interruption. Mean BP was 105/70 mmHg at the beginning of the exercise session, 120/70 mmHg in the middle, and 125/70 mmHg at the end.

On completion of the exercise program, while his pharmacologic treatment was unchanged, his symptoms were significantly reduced, presenting only one episode of angina at rest in the last four weeks and no angina while performing his activities. There was no change in clinical examination parameters.

The final CPET revealed a 60% VO _2_ peak increase, a 27-bpm angina threshold increase, and a decrease of the angina intensity from 7 to 5. There were no electrocardiographic abnormalities during exercise testing.

Patient continued exercising three times a week for the next two years after the end of the in-hospital program, with progressive improvement in symptoms and quality of life.

## Discussion

We reported an important clinical improvement of a patient with RA considered “no-option” after a 12-week individualized ECR program safely executed, despite aerobic exercise training had been performed above the ischemic/angina threshold obtained in CPT, reinforcing the significance of this adjuvant but essential part of clinical management of these patients.

ECR, although recommended in some guidelines, ^8^ is rarely applied to RA patients due to little evidence of safety, efficacy, and peculiarities to “the way to do it.” It was studied in this population for the first time by Asbury et al., ^6^ demonstrating an improvement in *Progressive Shuttle Walking Test* duration, anxiety, and angina perception in an eight-week rehabilitation program. Montenegro et al. ^7^ recently also showed the safety of exercising RA patients, since a 40-minute aerobic session, even in the presence of angina, did not increase us-troponin, therefore, did not provoke myocardial injury.

In the same way, Corre et al. ^10^ reported a case of a patient with RA who trained based on a 20-session protocol with high-intensity aerobic interval exercise, including intermittent ischemia, and presented an important improvement in angina symptoms and ischemic threshold. Such a strategy had been demonstrated to be safe previously ^11^ in 11 patients with ischemic heart disease.

Noteworthy, angina episodes during exercise training, when occurred, were at higher HR than the CPET angina threshold. This difference can be justified, in part, by different conditions imposed on a laboratory test and real-life training, such as the progressive load increment that occurs in CPET, added to the absence of warm-up and cool down-periods in the test. Thus, the CPET angina threshold HR is an important variable to guide the aerobic exercise prescription but not a limitation to real-life training.

Therefore, ECR was a safe and effective treatment strategy for this patient with RA, despite the occurrence of angina/ischemia during training, respecting the patient’s tolerability to symptoms and other warning clinical signs. It is important to remark that, as patients with RA present high-risk features for exercise-induced adverse cardiac events. ^1 , 12^ Therefore, conditions presented previously as clinical stability, determination of individual clinical parameters (angina threshold and angina level tolerability) to prescribe and guide trainning, as well as supervised and monitored in-hospital ECR are necessary for introducing these patients to exercise-based cardiac rehabilitation programs.

Given the current evidence, it is important to begin considering ECR as part of the treatment of these patients. Furthermore, larger and randomized studies are needed to reinforce this indication through more high-quality evidence.
